# Evaluating Patterns of Injectate Spread After Rectus Sheath Block: A Cadaveric Dissection Study

**DOI:** 10.7759/cureus.34142

**Published:** 2023-01-24

**Authors:** Andres Rojas, Daniel T McMillan, Jennifer D Allan, Monika Nanda, Chinwe Anumudu, Jeremy Armbruster, Maxwell Jolly, Justin Magin, Gisselle Maquoit, Stuart A Grant

**Affiliations:** 1 Anesthesiology, University of North Carolina at Chapel Hill School of Medicine, Chapel Hill, USA; 2 Anesthesiology, Duke University School of Medicine, Durham, USA

**Keywords:** rectus sheath block, cadaver dissection, ultra sound guided nerve block, chronic and acute pain management, regional anesthesia and chronic pain

## Abstract

Introduction: This cadaveric dye study assesses the effect of volume and number of injections on the spread of solution after ultrasound-guided rectus sheath injections. In addition, this study evaluates the impact of the arcuate line on solution spread.

Materials and methods: Ultrasound-guided rectus sheath injections were performed on seven cadavers on both sides of the abdomen, for a total of 14 injections. Three cadavers received one injection of 30 mL of a solution consisting of bupivacaine and methylene blue at the level of the umbilicus. Four cadavers received two injections of 15 mL of the same solution, one midway between the xiphoid process and umbilicus and one midway between the umbilicus and pubis.

Results: Six cadavers were successfully dissected and analyzed for a total of 12 injections, while one cadaver was excluded due to poor tissue quality that was inadequate for dissection and analysis. There was a significant spread of solution with all injections caudally to the pubis without limitation by the arcuate line. However, a single 30 mL injection showed inconsistent spread to the subcostal margin in four of six injections, including in a cadaver with an ostomy. A double injection of 15 mL showed consistent spread from xiphoid to pubis in five of six injections, except in a cadaver with a hernia.

Conclusions: Injections deep to the rectus abdominis muscle, using the same technique as an ultrasound-guided rectus sheath block, achieve spread along a large and continuous fascial plane without limitation by the arcuate line and may provide coverage of the entire anterior abdomen. A large volume is necessary for complete coverage and spread is improved with multiple injections. We suggest that two injections with a total volume of at least 30 mL per side may be needed to achieve adequate coverage in the absence of preexisting abdominal abnormalities.

## Introduction

The rectus sheath block has been traditionally used to provide analgesia for midline abdominal procedures. It was originally described as a blind needle insertion technique with the operator feeling fascial “pops.” The needle endpoint was described as within the rectus sheath, behind the muscle [1]. Ultrasound has allowed for more precision with the rectus sheath block, and it is now performed posterior to the rectus abdominis muscle but anterior to the posterior rectus sheath [2]. Historically, the extent of infraumbilical coverage and local anesthetic spread has been thought to be limited due to the presence of the arcuate line, where the posterior sheath of the rectus muscle ends and merges with the anterior sheath. Injecting below the arcuate line has even been discouraged due to the increased risk of peritoneal injection given the absence of the posterior rectus sheath. This claim continues even with the use of ultrasound. A recent review of abdominal wall blocks specifically mentions incisions such as the Pfannenstiel for obstetric surgery when discussing the rectus sheath block [3]. The authors argue that, since the posterior sheath ends at the arcuate line, there is no target plane in which local anesthetic can spread to cover this kind of incision. Additionally, there are limited studies demonstrating the optimal volume or dose of local anesthetic for this block [4-11].

There are limited published studies discussing local anesthetic dosing for ultrasound-guided rectus sheath blocks. Volumes utilized range widely from 10 to 30 mL of local anesthetic per side in adults [4-11] and 0.1-0.5 mL/kg in pediatric populations [12-14]. Cornish et al. described a volume of 20 mL for surgically placed catheters but no rationale was provided for this choice [4]. Many studies have reproduced this dose with ultrasound-guided rectus sheath blocks. One prospective trial by Cho et al. used a larger volume of 30 mL, but it is difficult to interpret whether this improved analgesia, because there was no comparison to lower-volume blocks and the study, was conducted in laparoscopic surgery cases [5].

To better understand the ultrasound-guided rectus abdominis block, a cadaver dissection and dye study was conducted to evaluate if either a larger volume of local anesthetic than previously described or if multiple injections between the posterior rectus sheath and rectus abdominis muscle could improve the spread of solution of this technique. Additionally, we aimed to determine if the arcuate line impacts the spread of injectate below the umbilicus.

The preliminary results of this study were presented as a scientific abstract at the 47th Annual American Society of Regional Anesthesia and Pain Medicine Meeting in Las Vegas, NV, on March 31, 2022.

## Materials and methods

The investigators performed bilateral dissections on seven cadavers for a total of 14 injections, one on each side of the abdomen. Six of the cadavers had no obvious prior abdominal surgery. One of the cadavers (Cadaver 1) had an enterostomy on the right side of the abdomen. Five cadavers used were embalmed and stored at room temperature for 10 days prior to dissection. One cadaver (Cadaver 2) was freshly frozen and then stored at room temperature for 48 hours prior to dissection. One cadaver (Cadaver 7) was embalmed and stored at room temperature for three weeks prior to dissection. Cadaver demographic information is summarized in Table 1. The cadavers were donated and supported by the body donation program at the University of North Carolina at Chapel Hill School of Medicine. This study was exempt from the institutional review board, and appropriate permissions were obtained by the School of Medicine.

**Table 1:** Cadaver demographic information

Cadaver	Gender	Age	Race	Cause of Death	Preservation Method
1	Female	84	White	Carcinoma of terminal ileum	Embalmed
2	Male	85	White	Congestive heart failure	Fresh frozen
3	Female	90	White	Dementia	Embalmed
4	Male	92	White	Cardiopulmonary arrest	Embalmed
5	Female	70	Black	Congestive heart failure	Embalmed
6	Female	87	White	Large cell lymphoma	Embalmed
7	Female	88	White	Pneumonia	Embalmed

**Figure 1:**
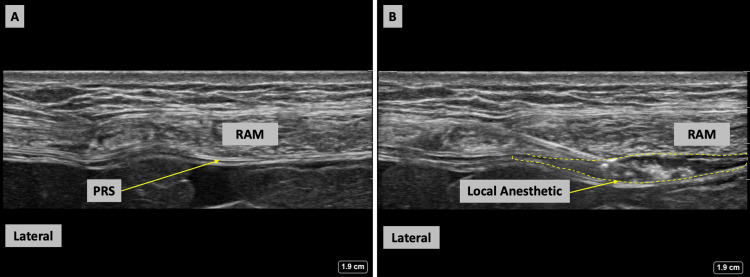
Ultrasound images of the rectus sheath injection technique (A) Before injection of local anesthetic, the RAM and the PRS are labeled. (B) The nerve block needle is advanced in-plane in a lateral to a medial trajectory and can be seen piercing the posterior border of RAM. Local anesthetic is deposited between the RAM and PRS and can be seen expanding this fascial plane. RAM = rectus abdominis muscle, PRS = posterior rectus sheath

The ultrasound-guided injections were performed using a high-frequency (15-6 MHz) linear transducer (SonoSite, Fujifilm, USA). With the cadaver positioned supine, the transducer was placed lateral to the midline in a transverse orientation at the level of the umbilicus. The external and internal oblique and the transversus abdominis muscles were observed laterally and used to identify the linea semilunaris and the posterior rectus sheath coursing under the rectus abdominis more medially. The ultrasound probe was translated caudally in search of the arcuate line. Then, with the ultrasound probe again at the level of the umbilicus, a 21G, 100 mm echogenic needle (SonoPlex, Pajunk, USA) was introduced in-plane, in a lateral to medial direction, through the rectus abdominis muscle until the needle tip was positioned between the posterior border of rectus abdominis and posterior rectus sheath. Six rectus sheath planes were injected with a single, 30-mL (28.5 mL of 0.25% bupivacaine with 1.5 mL of 10% methylene blue) injection, under ultrasound guidance at the level of the umbilicus (Figure 1). Necessary adjustments were made by retracting or advancing the block needle to maintain an appropriate position of the needle tip. The additional eight rectus sheath planes were injected utilizing a two-injection technique with two, 15-mL injections, for a total of 30 mL. One injection was performed halfway between the umbilicus and the xiphoid process, and the other was performed halfway between the umbilicus and the pubic symphysis. The injection sites were theorized to maximize spread over the entire plane between the rectus abdominis muscle and posterior rectus sheath. Given the variability in its location [3], the arcuate line was not used as a landmark to guide the infraumbilical injection.

Twenty minutes after the injections were performed, dissection was undertaken to visualize the space between the rectus abdominis muscle and posterior rectus sheath. With each cadaver laying supine, a vertical, midline incision was made with a scalpel through the skin and subcutaneous tissue. Sharp dissection was continued until the linea alba was identified. Dissection was continued carefully through the fascia on the medial border of the rectus abdominis muscle. Once the medial border of the rectus abdominis muscle was exposed, the anterior rectus sheath was incised vertically and the rectus abdominis muscle was then carefully reflected laterally to expose the posterior rectus sheath deep to the muscle with the anterior branches of the thoracic nerves lying on the fascia. The dissection was extended from xiphoid to pubic symphysis. Care was taken to preserve the perforating branches of the anterior cutaneous and muscular nerves piercing the posterior rectus sheath and rectus abdominis muscle. The posterior rectus sheath was separated from the rectus abdominis muscle laterally until the linea semilunaris was identified.

Due to the difficulty correlating clinically successful nerve blocks with the spread of blue dye, a high standard was chosen of only intense blue staining representing the successful spread of local anesthetic. Scant or no staining was determined to be failed spread. Only descriptive metrics were used; no quantitative measurement of stain intensity was utilized.

## Results

Under ultrasound examination, the rectus abdominis muscle and posterior rectus sheath were identifiable in all cadavers. The arcuate line was not identified in any of the dissections. The spread of local anesthetic was easily observed during injection extending in a cephalad and caudad direction. The extent of cephalo-caudad spread on ultrasound was not formally assessed.

At dissection, the fascial plane was easy to identify in 11 of the 14 dissections. In one cadaver (Cadaver 1), there was an ostomy on the right side with scarring and adhesions around the stoma. Cadaver 6 had poor tissue quality and the muscle broke down with handling, making a clean plane difficult to obtain. Cadaver 6 was not used in the assessment of fluid spread. In addition, although Cadaver 4 was easy to dissect, a hernia was identified on the lateral part of the right rectus abdominis muscle. Once the rectus abdominis muscle was reflected laterally to reveal the posterior rectus sheath, there was no identifiable arcuate line visible in any of the dissections. The posterior sheath of the rectus abdominis was found to be a continuous fascial layer permitting the spread of dye from the costal margin to the pubic symphysis except in the presence of a surgical scar or an abdominal wall hernia.

**Figure 2:**
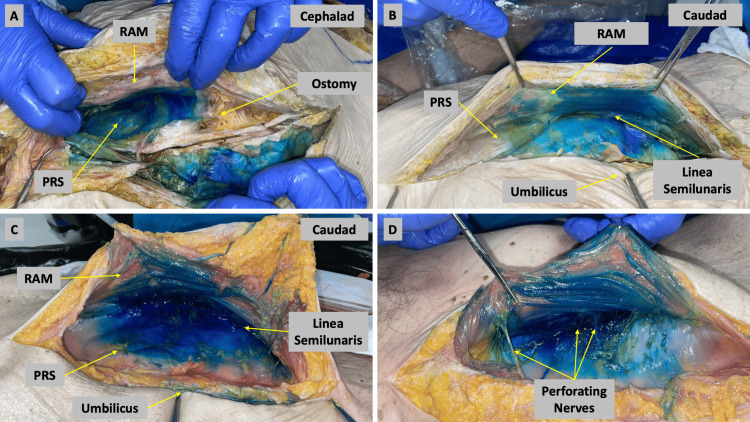
Images of the dissection of Cadavers 1 and 2 after a single, 30 mL injection, with RAM, reflected off of PRS (A) Cadaver 1, right side: Consistent staining of the PRS can be observed extending caudally. Spread is limited in the cephalad direction by the presence of an ostomy. (B) Cadaver 1 left side: Consistent dye spread is again observed caudally but is limited in the cephalad direction. Spread is also seen extending laterally to the linea semilunaris. (C) Cadaver 2, right side: Dye is seen spreading caudally throughout the dissection but less so in the cephalad direction. As in the other cadaver, dye spread is seen laterally as far as the linea semilunaris. (D) Dissection of Cadaver 2 showing the perforating branches of the rectus sheath plexus. RAM = rectus abdominis muscle, PRS = posterior rectus sheath

A single injection of 30 mL at the level of the umbilicus was performed and dissected successfully six times (Cadavers 1-3). Around 30 mL of injectate spread extensively from the site of injection but did not consistently cover the entire rectus sheath from the pubis to the costal margin. The spread caudally to the pubis was strong and consistent but the spread cephalad to the xiphoid and costal margin was not. Cadaver 1 had a preexisting stoma around the level of the T9 dermatome and this restricted any spread of dye cephalad beyond that point (Figure 2). The perforating branches of the rectus sheath plexus were also stained, though it was challenging to conserve these nerves throughout our dissections and evaluations (Figure 2D).

**Figure 3:**
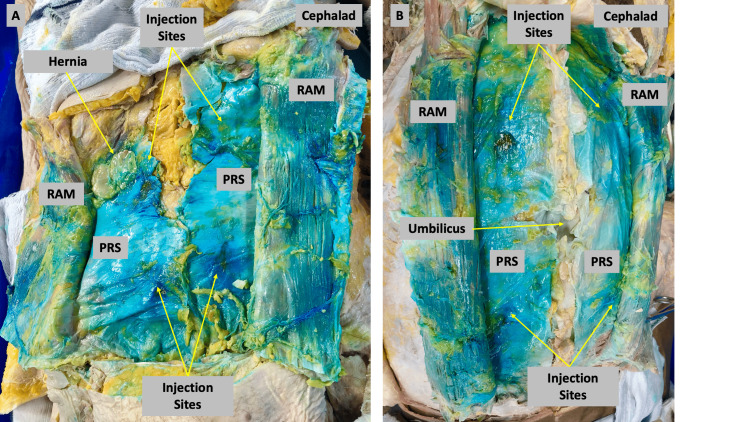
Dissection of Cadavers 4 and 5 after two 15 mL injections on both sides of the abdomen, one halfway between the umbilicus and the xiphoid process and the second halfway between the umbilicus and the pubis (A) Cadaver 4: bilateral dissection with RAM reflected off of PRS. The right side displays intense staining in the caudal direction. However, there is a hernia in the right upper quadrant of RAM that limits the cephalad spread of dye. The left side displays intense staining from the xiphoid to the pubis. (B) Cadaver 5: Both sides demonstrate significant staining at both sites of injection and in the cephalad and caudad directions. Staining around the level of the umbilicus is less intense but still significant. The left side displays staining that is slightly less intense than the right side overall, but the extent of spread is the same. RAM = rectus abdominis muscle, PRS = posterior rectus sheath

**Figure 4:**
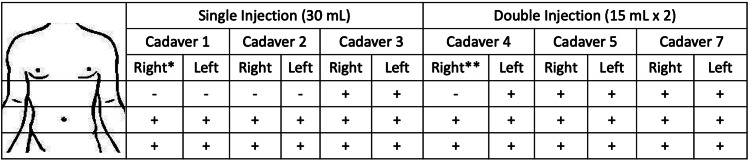
Summary of dissection results Cadavers 1-3 received a single 30 mL injection on both sides of the abdomen, for a total of six injections. Cadavers 4, 5, and 7 received two 15 mL injections, for a total of six injections. A "+" denotes strong or intense staining, while a "-” denotes scant to no staining. The spread of dye was consistent periumbilically and infraumbilically to the pubic bone in all dissections. However, the double injections showed a more consistent spread to the subxiphoid area, except in cadaver 4, which was found to have an abdominal wall hernia on the right side. *Ostomy, **Hernia

A double injection of 15 mL above and 15 mL below the umbilicus was performed eight times and six were successfully dissected (Cadavers 4, 5, and 7). Cadaver 4 had an abdominal wall hernia which restricted cephalad spread. A double injection of 15 mL demonstrated spread from the pubis to the xiphoid in all other injections. These injections demonstrated strong staining at the sites of injection that extended in a cephalad and caudad direction. Staining at the level of the umbilicus was less intense than at the injection site but was still significant (Figure 3). The results of the dissections are summarized in Figure 4.

## Discussion

The rectus sheath block is frequently used for postoperative analgesia after abdominal surgery. However, the evidence for the block’s efficacy, necessary volume, and dosing are lacking [4-11]. Previous authors have also suggested that the arcuate line prevents the caudad spread of local anesthetics and limits the utility of this block for low abdominal incisions [3]. This dissection dye study showed that an injection between the rectus abdominis muscle and posterior rectus sheath, a technique commonly used for ultrasound-guided rectus sheath blocks, allows for the spread of solution in a large, continuous space in both a cephalad and caudad direction. Of note, we observed no impedance in caudad spread by the arcuate line. This study suggests that spread is enhanced by a large volume of at least 30 mL in divided injections and is limited by the presence of abnormalities in the abdominal wall such as surgical scarring and hernias.

In the review of the pertinent anatomy, the nerve targets of this block are the anterior cutaneous and muscular branches of the ventral rami of thoracolumbar roots T6-L1. The T9-L1 segmental nerves form an intricate plexus of communicating branches within the transversus abdominis plane. The branches subsequently converge and pierce the linea semilunaris, entering the rectus sheath in the space between the posterior rectus sheath and rectus abdominis. These nerves then undergo further branching to form the rectus sheath plexus. Branches of this plexus pierce the rectus abdominis muscle and provide motor innervation to the rectus abdominis and sensory innervation to the anteromedial abdomen [15]. Given the complexity, branching, and overlap of the multiple nerve segments that innervate the abdominal wall, a large degree of spread is required for complete coverage of the anterior abdomen and optimal analgesia.

The spread of solution with the volumes utilized was extensive along the posterior rectus sheath in the absence of abdominal wall derangements, though solution spread less readily in the cephalad direction. This was a notable finding, as rectus sheath blocks are often utilized for upper abdominal procedures and it is caudad spread that is thought to be limited by the presence of the arcuate line. Possible factors include the role of the costal margin, upper abdominal and lower thoracic cavity contents (liver, spleen, diaphragm), and biomechanical limitations that, to our knowledge, have not been described. Elsharkawy et al. discussed this concept when describing the mechanism of action of fascial plane blocks [16]. It is possible that there are differences in the spread in live subjects compared to cadavers due to different biomechanical conditions.

The arcuate line is frequently cited as a barrier to caudad spread after injection of local anesthetic within the rectus sheath [3]. The location of the arcuate line is traditionally described as variable and exists somewhere between the umbilicus and the pubic bone. Of note, the arcuate line was not identified in this project in any cadaver on examination by ultrasound or dissection. There are multiple possible explanations for this result. Monkhouse et al. showed that the arcuate line may be as high as the umbilicus and as low as within a few centimeters of the pubic bone, and is rarely symmetric within the same specimen [17]. This significant variability may help explain why the arcuate line was not identified, especially with the small sample size utilized for this study. Additionally, minimal muscle mass in elderly cadavers made it challenging to identify the arcuate line as the posterior rectus sheath transitioned from posterior to anterior to the rectus abdominis. However, the posterior rectus sheath and fascia transversalis were identified with ultrasound in all of the dissections, so this would not completely explain the apparent absence of the arcuate line. Furthermore, the arcuate line can be found where the inferior epigastric vessels perforate the rectus sheath, and the lack of pulsatile and/or compressible vessel-like structures under ultrasound visualization may have contributed. Given the subtle nature of the arcuate line, it is possible that intense staining from the dye solution may have obscured the line. Finally, while the investigators who performed the dissections had experience with cadaveric dissection as part of their medical training, having an expert anatomist could have reduced variability caused by the distortion of tissues and may have facilitated the identification of the arcuate line. Nonetheless, the heterogeneity in the location of the arcuate line and the findings of this study suggest that the line may not be a dense, discrete structure that limits the spread of solution like previously suggested. There appears to be a continuation of the posterior rectus sheath on which solution can spread beyond the arcuate line, unlike traditionally described [3].

As of the writing of this manuscript, there are no studies evaluating either the volume necessary for adequate spread or the extent of solution spread in human cadaveric dissections after injection between the rectus abdominis and posterior rectus sheath. Many authors have evaluated the efficacy of rectus sheath blocks in different clinical settings [1,6-8]. There have also been studies published evaluating the spread of injectate under ultrasound [10]. Seidel et al. performed cadaveric dissections to assess the staining of target nerves with a medial-to-lateral approach compared to a lateral-to-medial injection but did not measure cephalad to caudad spread [11]. Many recent publications have evaluated spread after an ultrasound-guided rectus sheath injection in animal models, with differing results [18,19]. The results of this project provide needed guidance for optimizing the volume needed for ultrasound-guided rectus sheath blocks.

A volume of 20 mL is often used for this block [5], but the results of this study suggest that this volume is likely insufficient to achieve complete coverage of the anterior abdomen. As is described here, even 30 mL as a single injection may not offer complete coverage in every case. A double injection of at least 15 mL per injection bilaterally may more consistently offer coverage from xiphoid to pubis and could improve the clinical efficacy of this block for large midline incisions. In addition, the spread was significantly affected by preexisting abdominal derangements such as ostomies and hernias. A careful history that includes a review of a patient’s surgical history is important in predicting the benefits and limitations of a rectus sheath block.

Our study was limited by a small sample size as only seven cadavers were available to use for the dissection and only six yielded adequate dissections. As mentioned previously, all cadavers were elderly subjects with minimal muscle mass, and adequate dissection was impossible in one cadaver due to delicate and easily disrupted tissues. Additionally, one cadaver was freshly frozen while the others were embalmed, potentially introducing further variability in the dye spread, despite this not being detected. The injections were performed by operators with experience in regional anesthesia but the injection pressure and speed were not controlled, which may have introduced variability in solution spread. Debate continues regarding the use of dye as a proxy for local anesthetic spread. However, in using methylene blue (which has a molecular weight similar to local anesthetics) between two dense fascial planes, we did observe differences in spread using different volumes of solution, especially in cephalad spread. Finally, the spread of local anesthetic may be different in living subjects and common clinical scenarios. The spread of solution is likely to be affected depending on the timing of injection, type of incision, and surgical procedure. As demonstrated here, any derangements in the abdominal wall have a significant effect on the spread.

## Conclusions

The results of this project suggest that an injection between the rectus abdominis and posterior rectus sheath, like that used in ultrasound-guided rectus sheath blocks, results in a spread of local anesthetic that may provide adequate coverage of the anterior abdomen, including low abdominal incisions. Local anesthetic spreads within a large, continuous, fascial plane without limitation by the arcuate line, which, notably, was not identified either by ultrasound or dissection. A large volume is necessary for adequate coverage and multiple injections demonstrated better results. We propose that two injections with a total volume of at least 30 mL per side are likely to achieve complete coverage of the anterior abdomen in the absence of previous abdominal abnormalities such as ostomies, hernias, and surgical scarring. These findings also highlight the importance of obtaining a thorough clinical history to predict the benefits and limitations of this and other fascial plane blocks. Additional studies are needed to further evaluate this hypothesis and better define the required volume and dosing for this block in clinical practice.
